# High-Indium-Composition, Ultra-Low-Power GaAsSb/InGaAs Heterojunction Tunnel Field-Effect Transistors

**DOI:** 10.3390/mi17020149

**Published:** 2026-01-23

**Authors:** Yan Liu, Xiang Li, Dao-Hua Zhang, Meng-Qi Fan, Xiao-Ping Wang, Yun-Jiang Jin

**Affiliations:** 1 Shenzhen Pinghu Laboratory, Shenzhen 518111, China; 2 State Key Laboratory of Optoelectronic Materials and Technologies, School of Electronics and Information Technology, Sun Yat-Sen University, Guangzhou 510275, China

**Keywords:** GaAsSb/InGaAs heterojunction, parameter optimization, subthreshold swing, tunnel field-effect transistors (TFETs)

## Abstract

In this work, we report the first systematic examination of how the In composition in the intrinsic In_x_Ga_1-x_As layer and the p-type doping concentration in the p-type GaAsSb layer affect the device performance of side-gate p-GaAs_0.5_Sb_0.5_/i-In_x_Ga_1-x_As/n-In_0.53_Ga_0.47_As TFETs, using the technology computer-aided design (TCAD) simulations with a non-local band-to-band model. By tuning these two parameters, a moderate staggered alignment is achieved, enabling self-off operation at zero gate bias while maintaining high on-current. This tunability is an intrinsic and significant advantage of the p-GaAsSb/i-In_x_Ga_1-x_As heterojunction that has not been previously explored. It is found that the best device performance does not occur in the TFET with an In composition of 0.53 in the intrinsic layer, which is lattice-matched to the InP substrate, but rather occurs in the device with a higher In composition of around 0.59 in the InGaAs layer, which has been verified by experimental data to some extent. Optimal parameter combinations yield a minimum subthreshold swing of 13.51 mV/dec and an ON-state current of 35.39 μA/μm at V_DS_ = V_GS_ = 0.5 V due to the enhanced tunneling capability.

## 1. Introduction

For decades, the scaling of MOSFETs has driven continuous improvements in integrated circuit performance. With the approaching end of Moore’s law, power dissipation has become a primary constraint in further improving transistor density and computing performance. Conventional CMOS devices are fundamentally limited by the Boltzmann distribution of carriers, resulting in a minimum subthreshold swing (SS) of 60 mV/dec at room temperature [1]. To overcome this limitation, several steep-slope transistor concepts have been proposed, such as negative-capacitance field-effect transistors (NC-FETs) [2], Dirac-source FETs (DS-FETs) [3], and tunnel FETs (TFETs) [4,5,6,7,8]. While NC-FETs and DS-FETs rely on emerging materials like ferroelectrics or two-dimensional materials that remain challenging for large-scale fabrication and integration, TFETs exploit the band-to-band tunneling (BTBT) within conventional semiconductor systems, offering better compatibility with mature epitaxy and fabrication technologies [9]. The reduced SS of TFETs grants them a higher transconductance to current ratio than traditional FETs [10]. The higher output resistance offered by TFET-based designs significantly higher intrinsic voltage gain and higher maximum-oscillation frequency at low current levels [11]. Furthermore, studies have demonstrated that TFETs show great promise for various applications, including energy harvesting systems [12], biosensors [13], and Internet of Things (IoT) devices [14].

Among various TFET material candidates, the GaAsSb/InGaAs heterojunction is particularly promising because of its flexibly tunable type-II band alignment and high carrier transport properties [15,16]. By carefully adjusting the In composition in InGaAs, the conduction- and valence-band offsets can be engineered to obtain a moderate staggered alignment. This alignment provides a sufficient but not overly extreme offset to enhance tunneling probability. As a result, the device maintains strong on-current while remaining off at zero gate bias, avoiding the normally-on behavior. Meanwhile, the p-type doping in GaAsSb source controls the depletion width and further optimizes the tunneling probability, providing an additional degree of freedom for device design. This tunability is the true advantage of the GaAsSb/InGaAs TFETs; however, most of the previous simulations and experiments based on this heterojunction system focus on InP lattice-matched GaAs_0.5_Sb_0.5_/In_0.53_Ga_0.47_As only [15,17,18,19,20,21,22,23,24], which thus motivates us to investigate it thoroughly.

In this work, we perform comprehensive technology computer-aided design (TCAD) simulations using a non-local BTBT model on the side-gate p-GaAsSb/i-InGaAs/n-InGaAs p-i-n TFET structure, where the p-type GaAsSb serves as the source, the intrinsic InGaAs as the channel, and the n-type InGaAs as the drain. The influences of In composition and source doping are investigated to quantify their effects on the band alignment, tunneling current, and subthreshold characteristics. Furthermore, the p-GaAsSb/i-InGaAs/n-InGaAs TFETs have been fabricated and their device performance has been characterized. The results provide important guidance for optimizing III-V heterojunction TFETs toward manufacturable low-power applications.

## 2. Device Structure and Model Construction

**Figure 1:**
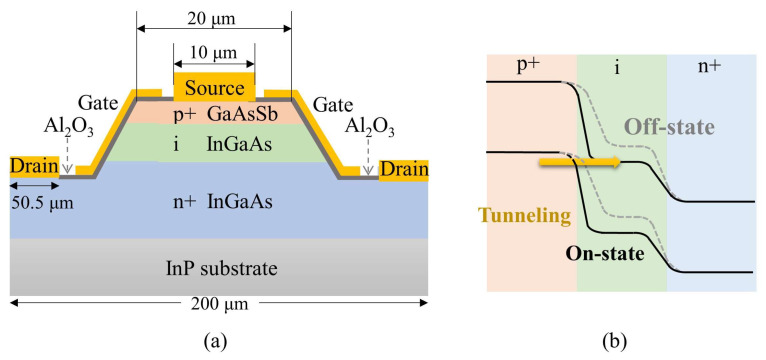
(**a**) Schematic of the p-GaAsSb/i-InGaAs/n-InGaAs heterojunction TFET with side gates, and (**b**) corresponding band diagram of the TFET.

The micrometer-sized device structure of the simulated p-GaAsSb/i-InGaAs/n-InGaAs TFET is illustrated in Figure 1a. On top of the semi-insulating InP substrate, the drain layer is 300 nm n-type In_0.53_Ga_0.47_As with a doping concentration of 1 × 10^19^ cm^−3^, followed by a 100 nm intrinsic In_x_Ga_1-x_As as the channel region. The source layer is a 60 nm p-type GaAs_0.5_Sb_0.5_. Both In_0.53_Ga_0.47_As and GaAs_0.5_Sb_0.5_ exhibit good lattice matching with the InP substrate [21], which helps to reduce interface defects and improve device performance. An Al_2_O_3_ interfacial oxide layer with a high dielectric constant of 9.3, as well as a sidewall gate structure, has been applied to enhance the electrostatic control over the p-GaAsSb/i-InGaAs tunneling junction. The band structure of this TFET, as illustrated in Figure 1b, will be carefully optimized to a moderate staggered alignment by adjusting the In composition in InGaAs. This optimization ensures that the device will maintain strong on-current while remaining off at zero gate bias.

The p-GaAsSb/i-InGaAs/n-InGaAs TFET model investigated in this work was constructed using commercial Silvaco TCAD software (Silvaco Victory Device V1.24.0). For the physical models, the non-local BTBT model is implemented to account for both forward and backward tunneling currents, as well as the continuous fluctuation of the bands of energy in the degenerately doped source TFET. The net current density for electrons with a longitudinal energy $E_{L}$ and a transverse energy $E_{T}$ can be given by

(1) $$J=\frac{q}{\pi ћ}\int \int T(E_{L})[f_{l}-f_{r}]\rho dE_{T}dE_{L}$$

where $f_{l}$ and $f_{r}$ are the Fermi–Dirac function using the quasi-Fermi level from the left and right side of the junction, respectively. ρ is the 2D density of states. The tunneling probability $T(E_{L})$ can be calculated by using the WKB approximation:

(2) $$T(E_{L})=\exp\left(-2\int_{x_{\mathrm{start}}}^{x_{\mathrm{end}}}k(x)dx\right)$$

where $x_{start}$ and $x_{end}$ are the starting and ending points of the tunneling paths, respectively. The k(x) can be expressed by

(3) $$k(x)=\frac{k_{e}k_{h}}{\sqrt{k_{e}^{2}k_{h}^{2}}}$$

where

(4) $$k_{e}(x)=\frac{1}{iћ}\sqrt{2m_{0}m_{e,tun}\left[E_{L}-E_{C}\left(x\right)\right]}$$

and

(5) $$k_{h}(x)=\frac{1}{iћ}\sqrt{2m_{0}m_{h,tun}\left[E_{V}\left(x\right)-E_{L}\right]}$$

where ћ is the reduced Planck’s constant, $m_{0}$ is the rest mass of the electron, and $m_{e,tun}$ and $m_{h,tun}$ are the relative tunneling mass for electrons and holes, respectively. *E_C_*(*x*) and *E_V_*(*x*) are the conduction band energy and valence band energy, respectively.

Other physical models, such as Shockley–Read–Hall (SRH) and Auger recombination, energy bandgap narrowing (BGN), Fermi–Dirac Statistics, and electric-field-dependent and concentration-dependent mobility models, are also applied. The SRH recombination rate can be achieved as

(6) $$R_{\mathrm{SRH}}=\frac{np-n_{\mathrm{ie}}^{2}}{\tau_{p}n+\tau_{n}p}$$

where $\tau_{n}$ and $\tau_{p}$ are the electron and hole lifetime, respectively. n and p are the electron and hole concentrations, and $n_{\mathrm{ie}}$ is the effective intrinsic concentration. Accounting for low-field and transverse-field effects on the mobility, the carrier mobility μ is calculated by Matthiessen’s rule. Moreover, as carriers are accelerated in an electric field, their velocity will begin to saturate when the electric field magnitude becomes significant. The field-dependent carrier mobility can be defined by Caughey and Thomas’ expressions [25], which provide a smooth transition between low-field and high-field behavior

(7) $$\mu =\mu_{0}{\left[1+{\left(\frac{\mu_{0}\left|E_{∥}\right|}{V_{\mathrm{sat}}}\right)}^{\beta }\right]}^{-1/\beta }$$

where $\left|E_{∥}\right|$ is the magnitude of the parallel electric field, $V_{\mathrm{sat}}$ is the carrier saturation velocity, $\mu_{0}$ is the low-field mobility, and the low-field electron and hole mobilities of In_x_Ga_1-x_As can be given by the following interpolation formulas

(8) $$\mu_{n0}=\left[33000x+8000\times \left(1-x\right)\right]{\left(\frac{T_{L}}{300K}\right)}^{-3/2}$$

and

(9) $$\mu_{p0}=\left[150x+400\times \left(1-x\right)\right]{\left(\frac{T_{L}}{300K}\right)}^{-3/2}$$

Specifically, for GaAsSb and InGaAs, the main material parameters are already included in the database of the software, and the CUBIC35 Model is considered, which allows the consistent modeling of the energy bandgaps, electron affinity, conduction band density of states, valence band density of states, and relative dielectric permittivity of III-V binary and ternary compounds using interpolation formulae. The dependence on lattice temperature is also included. Although the interface traps are known to impact the device performance of TFETs, the interface states only near the conduction band in the channel layer can affect the electrical characteristics of n-TFETs (electron tunneling dominates). Furthermore, their effects on the TFETs are smaller than those on the normal MOSFETs [17]. Consequently, to simplify the discussion in this work, only the tunneling mechanism is incorporated into the simulation, while other defect-associated currents and interface states are not considered.

## 3. Results and Discussion

### 3.1. Simulation Results

**Figure 2:**
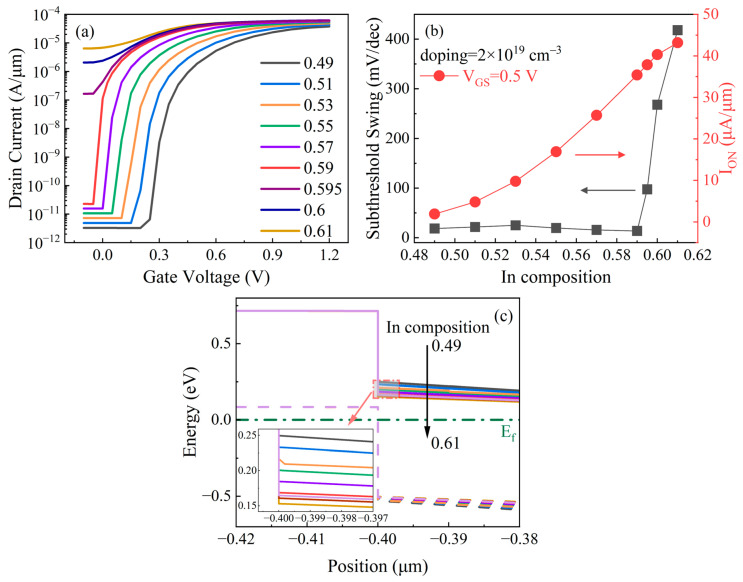
(**a**) Simulated transfer characteristics (I_DS_-V_GS_), (**b**) I_ON_ and minimum SS, and (**c**) part band diagram of the proposed p-GaAs_0.5_Sb_0.5_/i-In_x_Ga_1-x_As/n-In_0.53_Ga_0.47_As TFETs with different In compositions, the solid and dashed lines represent the energy of the conduction band and the valence band, respectively, and the dash-dotted line is the quasi-Fermi level.

Figure 2 shows the room-temperature transfer characteristics (I_DS_-V_GS_) and device performance of the p-GaAs_0.5_Sb_0.5_/i-In_x_Ga_1-x_As/n-In_0.53_Ga_0.47_As TFETs with different In compositions, under a fixed source doping level of 2 × 10^19^ cm^−3^. To align with next-generation logic technology, a low V_DS_ voltage of 0.5 V was set for conducting the device evaluation [15]. It can be found that the ON-state current (I_ON_) and OFF-state current (I_OFF_) increase with the In composition increases from 0.49 to 0.61. The increase in I_ON_ is mainly due to the bandgap shrinkage of i-In_x_Ga_1-x_As with a higher In composition [23]. A smaller bandgap directly reduces the offset between the p-GaAs_0.5_Sb_0.5_ valence-band and i-In_x_Ga_1-x_As conduction-band, and thus lowers the tunneling barrier, as can be observed in Figure 2c. Table 1 also lists the variation in main parameter values with In composition. This leads to an exponential increase in tunneling probability, which is described by Equations (2)–(5). Meanwhile, the increasing tunneling probability also impacts the undesirable tunneling under the Off-state bias, elevating the I_OFF_. In Figure 2b, when the In composition exceeds 0.59, the I_ON_ increases at a decelerated rate and tends to saturate, while the I_OFF_ rapidly increases. This leads to an abrupt switching transition, resulting in a degradation of the SS, which can also be observed in Figure 2b. When the In composition is less than 0.59, the SS of the studied TFETs is below 25 mV/dec with minor fluctuations and gradually decreases as the In composition increases from 0.53 to 0.59. Beyond this point, the SS rapidly rises to over 97 mV/dec. Regarding the optimal parameter, the TFET achieves the minimum SS of 13.51 mV/dec and an I_ON_ of 35.39 μA/μm at V_GS_ = 0.5 V, when the In composition is 0.59.

**Figure 3:**
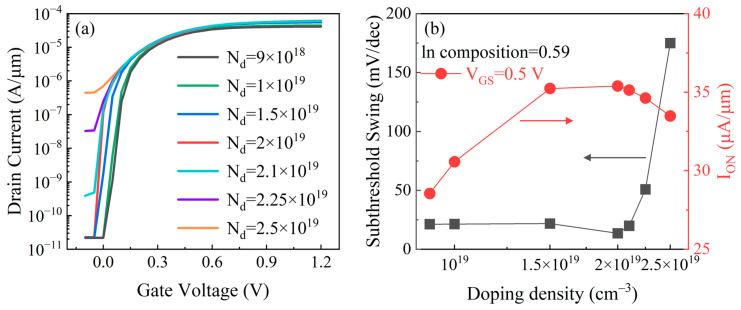
(**a**) Simulated transfer characteristics (I_DS_–V_GS_), and (**b**) I_ON_ and minimum SS of proposed p-GaAs_0.5_Sb_0.5_/i-In_0.59_Ga_0.41_As/n-In_0.53_Ga_0.47_As TFETs with different p-type doping concentrations of the GaAs_0.5_Sb_0.5_ layer.

Figure 3 presents the room-temperature transfer characteristics (I_DS_–V_GS_) and device performance of the p-GaAs_0.5_Sb_0.5_/i-In_x_Ga_1-x_As/n-In_0.53_Ga_0.47_As TFETs with different p-type doping concentrations of the GaAs_0.5_Sb_0.5_ layer at V_DS_ of 0.5 V. In Figure 3, as the doping concentration of GaAs_0.5_Sb_0.5_ varies from 9 × 10^18^ cm^−3^ to 2 × 10^19^ cm^−3^, I_ON_ continues to increase with doping concentration. This is because a higher doping level induces a steeper band profile at the tunneling junction, directly narrowing the tunneling barrier and thereby increasing the tunneling probability. When the doping reaches a higher level, such as 2.5 × 10^19^ cm^−3^, carrier degeneracy and electrostatic screening suppress further band bending, while bandgap narrowing reduces the built-in potential. As a result, the local electric field no longer improves, and the tunneling path reaches its minimum thickness, causing the I_ON_ to saturate. With regard to I_OFF_, as the doping concentration increases, it remains almost unchanged up to 2 × 10^19^ cm^−3^ and then degrades rapidly over 2.25 × 10^19^ cm^−3^. This behavior arises because, in the OFF-state, the band offset is large and the tunneling probability stays low even though the barrier becomes narrower. However, another effect occurs simultaneously: increasing the source doping also raises the valence-band edge and induces the bandgap narrowing. These two effects jointly reduce the tunneling barrier width and height. Once the doping reaches 2.25 × 10^19^ cm^−3^, this lowering effect becomes significant, leading to a rapid increase in band-to-band tunneling leakage and a pronounced rise in I_OFF_. Furthermore, in Figure 3b, due to the aforementioned variations in I_ON_ and I_OFF_, the SS of p-GaAs_0.5_Sb_0.5_/i-In_0.59_Ga_41_As/n-In_0.53_Ga_0.47_As TFETs gradually decreases with the doping concentration of the GaAs_0.5_Sb_0.5_ layer increasing from 9 × 10^18^ cm^−3^ to 2 × 10^19^ cm^−3^, and then steeply rises to 174.99 mV/dec when the doping concentration increases to 2.5 × 10^19^ cm^−3^.

### 3.2. Experiment Validation

**Figure 4:**
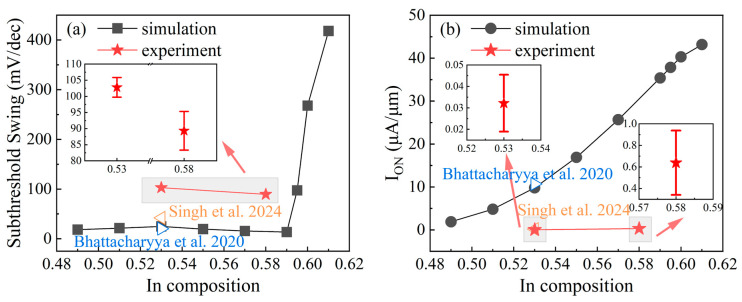
(**a**) Simulated and experimental minimum SS and (**b**) I_ON_ of p-GaAs_0.5_Sb_0.5_/i-In_0.59_Ga_0.41_As/n-In_0.53_Ga_0.47_As TFETs, hollow triangle and solid pentagram scatters represent the reported simulation results and our experimental data [21,26], respectively, and all the I_ON_ are corresponding to the V_GS_ = 0.5 V.

Figure 4 shows the simulated minimum SS and I_ON_ of the p-GaAs_0.5_Sb_0.5_/i-In_0.59_Ga_0.41_As/n-In_0.53_Ga_0.47_As TFET with the doping concentration of the GaAsSb layer of 2 × 10^19^ cm^−3^, and the reported simulation results and our experimental data are also given [21,26]. The reported minimum SS of GaAs_0.5_Sb_0.5_/In_0.53_Ga_0.47_As TFETs is in close agreement with our simulated results in Figure 4a, and the reported I_ON_ has an acceptable discrepancy relative to our simulated data in Figure 4b, which confirms the validity of our simulation. Moreover, previous works mostly choose the InP lattice-matched In_0.53_Ga_0.47_As as the channel material [15,17,18,19,20,21,22,23,24], because the lattice mismatch between different epitaxial layers and the substrate introduces strain [27], which relaxes by forming crystal defects at the interface and within the material itself. These defects act as trapping centers that severely degrade device performance. Although the lattice mismatch between In_0.59_Ga_0.41_As and InP is approximately 0.4% [28], the experimental results show that for a lattice mismatch of ±1% or less, the crystalline quality of epitaxial layers of InGaAs remains high with a thickness of 100 nm [29]. Therefore, the lattice-mismatch effects on the device performance are almost negligible. Our simulation results show that the p-GaAs_0.5_Sb_0.5_/i-In_x_Ga_1-x_As/n-In_0.53_Ga_0.47_As TFET achieves better device performance when the In composition is 0.59. Compared to the InP lattice-matched p-GaAs_0.5_Sb_0.5_/i-In_0.53_Ga_0.47_As/n-In_0.53_Ga_0.47_As TFET under the same doping density of GaAsSb layer, the minimum SS of p-GaAs_0.5_Sb_0.5_/i-In_0.59_Ga_0.41_As/n-In_0.53_Ga_0.47_As TFET is reduced by about 45.74%, and the I_ON_ (V_GS_ = 0.5 V) is increased by about 261.49%.

**Figure 5:**
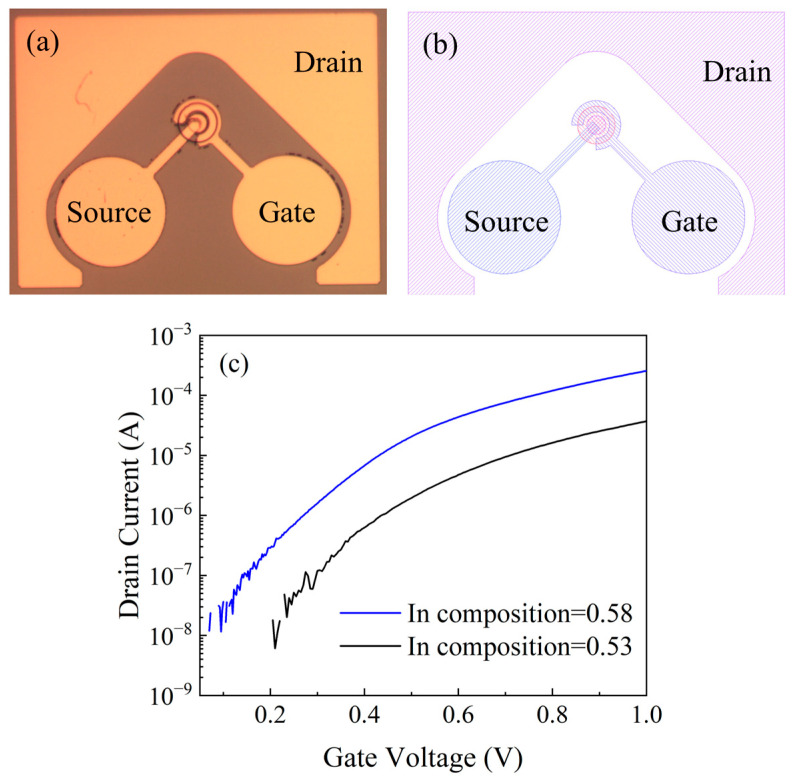
(**a**) The layout structure and (**b**) optical image of the actual device. (**c**) The transfer characteristics of p-GaAs_0.5_Sb_0.5_/i-In_x_Ga_1-x_As/n-In_0.53_Ga_0.47_As TFETs.

To further confirm this trend in the device’s performance, the epitaxial p-GaAs_0.5_Sb_0.5_/i-In_x_Ga_1-x_As/n-In_0.53_Ga_0.47_As structure was grown by molecular beam epitaxy (MBE) on semi-insulating InP substrate, and then fabricated into TFETs. The fabrication process starts with lithography and ICP-RIE etching to form the mesa. After that, an atomic-layer-deposition (ALD) Al_2_O_3_ was deposited as the gate oxide, followed by BOE etching of the oxide and source-/drain- metal deposition. Then the source and drain electrodes were patterned using the lift-off technique. At last, gate metal was deposited to complete the process. The layout structure, optical image, and transfer characteristics of actual devices are shown in Figure 5. The device structure of the simulation model is identical to that of the actual device. All the fabricated TFETs were measured from negative to positive bias under the same conditions. In our experiments, we intentionally selected one In composition lattice matched to the InP substrate and one In composition lattice mismatched to the InP to verify the validity of our model. The transfer characteristics of p-GaAs_0.5_Sb_0.5_/i-In_x_Ga_1-x_As/n-In_0.53_Ga_0.47_As TFETs are presented in Figure 5c; the I_ON_ of the device with higher In composition (0.58) is clearly better than that of the devices with lower Indium composition (0.53). Moreover, in Figure 4, according to the measurement results of several representative devices, the experimental minimum SS and I_ON_ of the p-GaAs_0.5_Sb_0.5_/i-In_0.58_Ga_0.42_As/n-In_0.53_Ga_0.47_As TFET are both better than those of the InP lattice-matched p-GaAs_0.5_Sb_0.5_/i-In_0.53_Ga_0.47_As/n-In_0.53_Ga_0.47_As TFET. Both the experimental and simulation results demonstrate that within the In composition range of 0.53 to 0.58, TFETs with a higher In composition exhibit superior device performance. This provides evidence that the moderate staggered alignment of the energy band in the p-GaAsSb/i-InGaAs heterojunction is likely to dominate the device performance, and the lattice mismatch does not constitute a primary factor influencing the minimum SS and I_ON_ of GaAsSb/InGaAs TFETs. Owing to the current limited experimental conditions, the experimental dataset will be expanded in our subsequent work to include results for a wider range of In compositions. In addition, a deviation can be observed between the simulation results and the experimental data. This is mainly because the idealized 2D device model utilized in the simulation is unable to capture the full complexity of the fabricated 3D structure. The material parameters used in the simulation do not perfectly match the properties of the actual fabricated materials, and the inherent process variations in fabrication also contribute to this deviation.

## 4. Conclusions

In summary, this work systematically investigates how In composition in intrinsic InGaAs and p-type doping concentration in GaAsSb affect the performance of side-gate p-GaAs_0.5_Sb_0.5_/i-In_0.59_Ga_0.41_As/n-In_0.53_Ga_0.47_As TFETs through TCAD simulations. By tuning the In composition, a moderate staggered band alignment is achieved, enabling self-off operation at zero gate bias while maintaining strong tunneling conduction. The optimized device performance is obtained at an In composition of approximately 0.59 and a GaAsSb p-type doping level of about 2 × 10^19^ cm^−3^, yielding a minimum SS of approximately 13.51 mV/dec, and an I_ON_ of 35.39 μA/μm at V_DS_ = V_GS_ = 0.5 V. Moreover, the device performance of the p-GaAs_0.5_Sb_0.5_/i-In_0.59_Ga_0.41_As/n-In_0.53_Ga_0.47_As TFET is notably superior to that of the InP lattice-matched p-GaAs_0.5_Sb_0.5_/i-In_0.53_Ga_0.47_As/n-In_0.53_Ga_0.47_As TFET, which has also been validated by experiments to a certain extent. These results offer the most promising and practically manufacturable material system for high-performance III-V TFETs, a clear design guideline. It is an important step toward large-scale, ultra-low-power electronic applications.

## Figures and Tables

**Table 1:** Variation in main parameter values with In composition.

Position (μm)	In Composition of i-In_x_Ga_1-x_As	Conduction Band Energy of i-In_x_Ga_1-x_As (eV)	Valence Band Energy of p-GaAs_0.5_Sb_0.5_ (eV)	Tunneling Barrier (eV)
−0.4	0.49	0.250	0.083	0.167
	0.51	0.233		0.150
	0.53	0.217		0.134
	0.55	0.200		0.117
	0.57	0.185		0.102
	0.59	0.169		0.086
	0.595	0.165		0.082
	0.6	0.161		0.078
	0.61	0.153		0.070

## Data Availability

The data presented in this study are available upon request from the corresponding author.

## References

[1] Shao Y., Pala M., Tang H., Wang B., Li J., Esseni D., del Alamo J.A. (2024). Scaled vertical-nanowire heterojunction tunnelling transistors with extreme quantum confinement. Nat. Electron..

[2] Cao W., Banerjee K. (2020). Is negative capacitance FET a steep-slope logic switch?. Nat. Commun..

[3] Baccichetti E., Marcon R., Esseni D. (2025). Minimum Subthreshold Swing in DS-FETs Based on Graphene and 3D Dirac Metals. IEEE Electron Device Lett..

[4] Soma U. (2025). Transistor Evolution: A Comprehensive Overview from TFT to TFET and Beyond. Proc. Natl. Acad. Sci. India Sect. A Phys. Sci..

[5] Tomioka K., Yoshimura M., Fukui T. Steep-slope tunnel field-effect transistors using III–V nanowire/Si heterojunction. Proceedings of the 2012 Symposium on VLSI Technology (VLSIT).

[6] Llorente C.D., Colinge J.P., Martinie S., Cristoloveanu S., Wan J., Le Royer C., Ghibaudo G., Vinet M. (2019). New prospects on high on-current and steep subthreshold slope for innovative Tunnel FET architectures. Solid-State Electron..

[7] Saeidi A., Rosca T., Memisevic E., Stolichnov I., Cavalieri M., Wernersson L.E., Ionescu A.M. (2020). Nanowire tunnel FET with simultaneously reduced subthermionic subthreshold swing and off-current due to negative capacitance and voltage pinning effects. Nano Lett..

[8] Borg B.M., Dick K.A., Ganjipour B., Pistol M.E., Wernersson L.E., Thelander C. (2010). InAs/GaSb heterostructure nanowires for tunnel field-effect transistors. Nano Lett..

[9] Allemang C.R., Anderson E.M., Gao X., Arose C., Mendez J.P., Weingartner T.A., Campbell D.M., Muhowski A.J., Lu T.-M., Misra S. (2025). Next-generation tunnel FETs: Exploring material perspectives and areal tunneling configurations. Mater. Quantum Technol..

[10] Rangasamy G., Zhu Z., Fhager L.O., Wernersson L.E. (2023). gm/I d g_m/I_d Analysis of vertical nanowire III–V TFETs. Electron. Lett..

[11] Rangasamy G., Zhu Z., Fhager L.O., Wernersson L.E. (2024). TFET circuit configurations operating below 60 mV/dec. IEEE Trans. Nanotechnol..

[12] Liu H., Li X., Vaddi R., Ma K., Datta S., Narayanan V. (2014). Tunnel FET RF rectifier design for energy harvesting applications. IEEE J. Emerg. Sel. Top. Circuits Syst..

[13] Reddy N.N., Panda D.K. (2021). A comprehensive review on tunnel field-effect transistor (TFET) based biosensors: Recent advances and future prospects on device structure and sensitivity. Silicon.

[14] Chander S., Sinha S.K., Chaudhary R. (2023). Prospects and challenges of different geometries of TFET devices for IoT applications. Nanosci. Nanotechnol.-Asia.

[15] Anam A., Amin S.I., Prasad D. (2024). Optimizing InGaAs/GaAsSb Staggered Bandgap U-Gate Line TFET with p+-Pocket Implant and Negative Capacitance for Enhanced Performance. IEEE Trans. Nanotechnol..

[16] Alian A., Kazzi S.E., Verhulst A., Milenin A., Pinna N., Ivanov T., Lin D., Mocuta D., Collaert N. Record 47 mV/dec top-down vertical nanowire InGaAs/GaAsSb tunnel FETs. Proceedings of the 2018 IEEE Symposium on VLSI Technology.

[17] Gotow T., Mitsuhara M., Hoshi T., Sugiyama H., Takenaka M., Takagi S. (2017). Effects of impurity and composition profiles on electrical characteristics of GaAsSb/InGaAs hetero-junction vertical tunnel field effect transistors. J. Appl. Phys..

[18] Yu T., Teherani J.T., Antoniadis D.A., Hoyt J.L. (2013). In_0.53_Ga_0.47_As/GaAs_0.5_Sb_0.5_ Quantum-Well Tunnel-FETs with Tunable Backward Diode Characteristics. IEEE Electron Device Lett..

[19] Convertino C., Zota C.B., Schmid H., Caimi D., Czornomaz L., Ionescu A.M., Moselund K.E. (2021). A hybrid III–V tunnel FET and MOSFET technology platform integrated on silicon. Nat. Electron..

[20] Yan Z., Li C., Guo J., Zhuang Y. (2019). A GaAs_0.5_Sb_0.5_/In_0.53_Ga_0.47_As heterojunction Z-gate TFET with hetero-gate-dielectric. Superlattices Microstruct..

[21] Bhattacharyya A., Chanda M., De D. (2020). GaAs_0.5_Sb_0.5_/In_0.53_Ga_0.47_As heterojunction dopingless charge plasma-based tunnel FET for analog/digital performance improvement. Superlattices Microstruct..

[22] Mohata D., Mookerjea S., Agrawal A., Li Y., Mayer T., Narayanan V., Liu A., Loubychev D., Fastenau J., Datta S. (2011). Experimental staggered-source and N+ pocket-doped channel III–V tunnel field-effect transistors and their scalabilities. Appl. Phys. Express.

[23] Smets Q., Verhulst A.S., El Kazzi S., Gundlach D., Richter C.A., Mocuta A., Collaert N., Thean A.V.-Y., Heyns M.M. (2016). Calibration of the effective tunneling bandgap in GaAsSb/InGaAs for improved TFET performance prediction. IEEE Trans. Electron Devices.

[24] Ohashi K., Fujimatsu M., Iwata S., Miyamoto Y. (2015). Body width dependence of subthreshold slope and on-current in GaAsSb/InGaAs double-gate vertical tunnel FETs. Jpn. J. Appl. Phys..

[25] Caughey D.M., Thomas R.E. (1967). Carrier mobilities in silicon empirically related to doping and field. Proc. IEEE.

[26] Singh S., Singh J. (2024). Design and estimation of GaAsSb/InGaAs hetero-junction double-dual gate vertical tunnel FET (HJ-VTFET) biosensor. J. Mater. Sci. Mater. Electron..

[27] Chu S.N.G., Macrander A.T., Strege K.E., Johnston W.D. (1985). Misfit stress in InGaAs/InP heteroepitaxial structures grown by vapor-phase epitaxy. J. Appl. Phys..

[28] Van de Walle C.G. (1989). Band lineups and deformation potentials in the model-solid theory. Phys. Rev. B Condens. Matter.

[29] Bennett B.R., del Alamo J.A. (1993). Mismatched InGaAs/InP and InAlAs/InP heterostructures with high crystalline quality. J. Appl. Phys..

